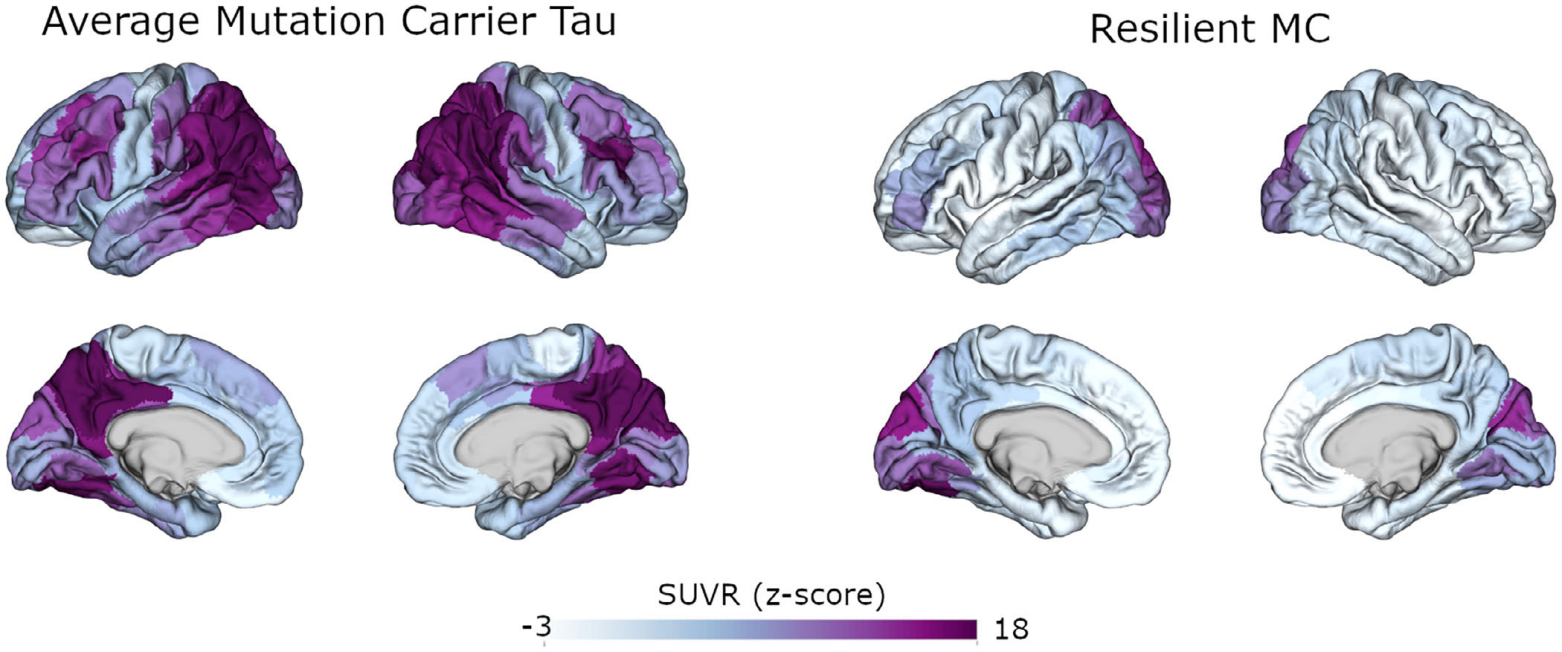# Task fMRI activity in an Autosomal Dominant mutation carrier demonstrating extreme resilience

**DOI:** 10.1002/alz70862_109779

**Published:** 2025-12-23

**Authors:** Brian A. Gordon, Diana A Hobbs, Mei Murphy, Edita Sabaredzovic, Lucas Paulson, Tyler M Blazey, Beau Ances, Nelly Joseph‐Mathurin, Jorge J. Llibre‐Guerra, Natalie S Ryan, Alan E. Renton, Eric McDade, Randall J. Bateman, Tammie L.S. Benzinger

**Affiliations:** ^1^ Washington University School of Medicine, Saint Louis, MO USA; ^2^ Washington University School of Medicine, St. Louis, MO USA; ^3^ Washington University in St. Louis School of Medicine, St. Louis, MO USA; ^4^ UK Dementia Research Institute at UCL, London UK; ^5^ Icahn School of Medicine at Mount Sinai, New York, NY USA; ^6^ Washington University St. Louis School of Medicine, St. Louis, MO USA; ^7^ Washington University in St. Louis, St. Louis, MO USA

## Abstract

**Background:**

Individuals with autosomal dominant Alzheimer Disease (ADAD) develop dementia at a relatively young age (late 30s‐ early 50s). We have conducted a deep phenotyping of highly resilient mutation carrier (rMC) carrying the presenilin 2 p.Asn141Ile mutation who has remained non‐demented more than two decades beyond their expected onset of dementia.

**Methods:**

Dementia status was determined using the clinical dementia rating (CDR). Tau PET imaging was performed using flortaucipir and amyloid imaging was performed using PiB. For visualization SUVRs were also converted to z‐scores relative to non‐carrier controls. In addition, the rMC underwent two task paradigms from the Human Connectome Project Aging (HCP Aging) study. These included a visual checkerboard test as well as a face‐name paired‐associate memory task.

**Results:**

The rMC remained cognitively unimpaired (CDR=0). The rMC demonstrated highly elevated levels of amyloid in a global summary measure (SUVR ∼3.6). The spatial distribution of amyloid was typical for ADAD. The rMC had a highly atypical pattern of tau pathology (Figure 1); Tau pathology was primarily constrained to visual cortex with pathology asymmetrically lateralized to the left hemisphere.

**Conclusions:**

The high levels of amyloid indicate a preserved penetrance of the mutation. The atypical tau pathology, greatly limited spread relative to expected patterns, suggests why overall cognition is preserved. However it is unknown if the cortical areas with high tau are truly resilient to pathology. It is possible that the tissue is impaired, but overt cognitive deficits are undetected due to the unilateral presentation largely limiting impairment to one hemisphere. The analysis of the task fMRI data will directly inform us as to the health of the underlying tissue. This will help differentiate if the resilience is tied to a reduced toxicity or biological resistance to the tau pathology or the reduced spread of pathology. Differentiating these outcomes will be incredibly important to interpreting mechanisms that infer resilience, even with strong genetic drivers of pathology.